# The Majority of Genotypes of the Virulence Gene *inlA* Are Intact among Natural Watershed Isolates of *Listeria monocytogenes* from the Central California Coast

**DOI:** 10.1371/journal.pone.0167566

**Published:** 2016-12-01

**Authors:** Lisa Gorski, Craig T. Parker, Anita S. Liang, Samarpita Walker, Kelly F. Romanolo

**Affiliations:** Produce Safety and Microbiology Research Unit, USDA, Agricultural Research Service, Albany, CA; UNITED STATES

## Abstract

Internalin A is an essential virulence gene involved in the uptake of the foodborne pathogen *Listeria monocytogenes* into host cells. It is intact in clinical strains and often truncated due to Premature Stop Codons (PMSCs) in isolates from processed foods and processing facilities. Less information is known about environmental isolates. We sequenced the *inlA* alleles and did Multi Locus Variable Number Tandem Repeat Analysis (MLVA) on 112 *L*. *monocytogenes* isolates from a 3-year period from naturally contaminated watersheds near a leafy green growing area in Central California. The collection contained 14 serotype 1/2a, 12 serotype 1/2b, and 86 serotype 4b strains. Twenty-seven different *inlA* alleles were found. Twenty-three of the alleles are predicted to encode intact copies of InlA, while three contain PMSCs. Another allele has a 9-nucleotide deletion, previously described for a clinical strain, indicating that it is still functional. Intact *inlA* genes were found in 101 isolates, and 8 isolates contained the allele predicted to contain the 3-amino acid deletion. Both allele types were found throughout the 3-year sampling period. Three strains contained *inlA* alleles with PMSCs, and these were found only during the first 3 months of the study. SNP analysis of the intact alleles indicated clustering of alleles based on serotype and lineage with serotypes 1/2b and 4b (lineage I strains) clustering together, and serotype 1/2a (lineage II strains) clustering separately. The combination of serotype, MLVA types, and *inlA* allele types indicate that the 112 isolates reflect at least 49 different strains of *L*. *monocytogenes*. The finding that 90% of environmental *L*. *monocytogenes* isolates contain intact *inlA* alleles varies significantly from isolates found in processing plants. This information is important to public health labs and growers as to the varieties of *L*. *monocytogenes* that could potentially contaminate fresh produce in the field by various means.

## Introduction

*Listeria monocytogenes* is a hardy, Gram-positive, saprophytic bacterium that is ubiquitous in the environment and causes systemic foodborne listeriosis. Between 2000 and 2008 there were an estimated 1600 cases of listeriosis per year in the U.S. with a hospitalization rate of 94% and a fatality rate of 19% [1]. In immunocompromised individuals, listeriosis manifests in a variety of ways including septicemia, meningitis, liver damage, and spontaneous abortion or preterm labor in pregnant women.

The 800 amino acid protein Internalin A, encoded by the *inlA* gene, is an essential virulence factor for *L*. *monocytogenes*, and facilitates entry of the pathogen into epithelial cells via interaction with E-cadherin [2]. Clinical isolates of *L*. *monocytogenes* carry an intact *inlA* gene. In surveys of foods and food processing environments for *L*. *monocytogenes* strains, about 40–50% of them carry truncated *inlA* genes because of mutations that result in premature stop codons (PMSCs) [3–7]. The PMSCs result in virulence attenuated strains that are reduced in their capacity to invade host cells [6, 8]. Several characterizations of *inlA* alleles from food and food-processing plant isolates of *L*. *monocytogenes* have been published, and several types of PMSCs have been described [4–12]. There is less information on *inlA* alleles in strains from environmental origins, but those published studies indicate that *inlA* alleles predominantly are intact both in healthy ruminants [8], and farm animals and their environments [10, 13].

There are 13 serotypes of *L*. *monocytogenes*, but the overwhelming majority of human illness (> 90%) is caused by serotypes 4b, 1/2b, and 1/2a, with serotype 4b being most common [14, 15]. *L*. *monocytogenes* is also subtyped into genetic lineages determined originally by Pulse Field Gel Electrophoresis, ribotyping, virulence gene variation, and DNA microarrays [12, 16–18], which show relationships among serotypes. Serotypes 4b and 1/2b, as well as serotypes 3b, 4d, and 4e, cluster in Lineage I, which contains the majority of human clinical strains [12]. Strains of serotype 1/2a, 1/2c, 3a, and 3c are clustered in Lineage II, which are found often in contaminated foods as well as being implicated in some outbreaks [19]. Lineages III and IV contain strains of serotypes 4a and 4c, along with some serotype 4b strains, and are often isolated from ruminants [20, 21]. Several studies conclude that strains with PMSCs are found more often among Lineage II rather than Lineage I [7, 8, 13]. Interestingly serotype 1/2a (Lineage II) strains are the serotype most often isolated from foods and food processing plants [14, 22].

There are location-specific differences in the prevalent *L*. *monocytogenes* serotypes found in environmental and watershed surveys. Surveys conducted in Eastern Canada and the Northeastern United States isolate serotype 1/2a (Lineage II) strains more often than serotype 4b and 1/2b (Lineage I) strains from soil and watersheds [23–25]. However, in an ongoing 5-year survey of water and sediment in public access watersheds in Central California there is a 43% total incidence of *L*. *monocytogenes*, and the serotype distribution of the isolates is serotype 4b (85%), serotype 1/2a (6.7%) and serotype 1/2b (5.4%), with greater intraserotype diversity found through Multi-Locus Variable Number Tandem Repeat analysis (MLVA) analysis [26, 27]. Sample sites consist of water and sediment in streams, ponds, lakes, and rivers. These water sources can become contaminated with pathogens from wildlife, sewage, and agricultural runoff, and while not used for crop irrigation in the region, these waters can serve as reservoirs for pathogens and contaminate crops during flood events and transfer by wildlife [26, 28–30].

While PMSCs in *inlA* are more commonly found among serotype 1/2a (Lineage II) strains, we wanted to know what type, if any, PMSCs were present in strains from our survey, and to determine the diversity of intact or mutant *inlA* alleles that persist in this Central California environment. We selected 112 *L*. *monocytogenes* water/sediment isolates from our watershed survey of serotypes 1/2a, 1/2b, and 4b that were isolated from 2012–2014 to determine the diversity of *inlA* genotypes, and what alleles persisted in those watersheds over time.

## Materials and Methods

### Watershed sampling, strain isolation, and differentiation

**Fig 1:**
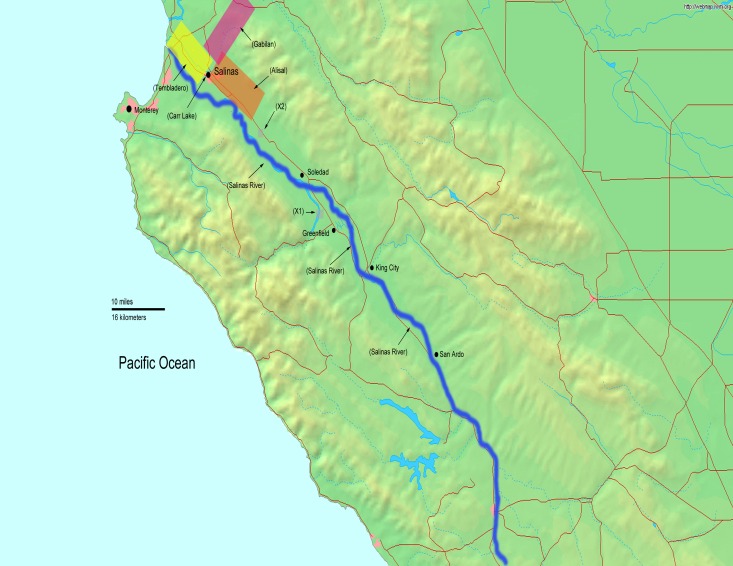
Sampling Area. The watersheds are indicated by overlaid colors on the green map background. Sampling sites not associated with watersheds along the waterways are indicated with X1 and X2 on the map. Sampling locations were grouped into watersheds to allow statistical analysis as described [26]. Watershed areas are shaded labeled. Salinas River watershed is indicated with the blue line along the Salinas River; Tembladero is the yellow box; Gabilan is the pink box; and Alisal is the orange box. The Carr Lake watershed is between the Tembladero and Alisal watersheds within the city of Salinas. The base layer of the map is from the DEMIS Mapserver. (http://www.demis.nl/home/pages/wms/demiswms.htm), which are public domain. DEMIS World Map Server generates maps using public domain data with no usage restrictions.

Sampling sites and enrichment, isolation, confirmation, and serotyping of *L*. *monocytogenes* strains were described [26, 27, 31]. Briefly, Moore swabs constructed of cheesecloth wrapped and tied with fishing wire were deployed into water sources (lakes, ponds, stream, rivers) with the wire then fastened to vegetation or rocks on the shore so that the swab remained present for 24 hours. No specific permissions were required for sampling because all locations were on public lands. These studies did not affect endangered or protected species. After exposure, the swabs were collected, stored on ice, transported to the lab, and used for enrichment cultures. A 500-square mile region of Central California along the Salinas River between the Gabilan Mountains and the Pacific Ocean was sampled bimonthly. Sampling sites included several sites along the Salinas River as well as streams, creeks, and ponds. Swabs were deployed at the same sites each sampling time. The area was sampled bimonthly, and comprised approximately 30 sites in a 500 square mile region that were grouped into 5 watersheds and 3 locations independent of any watershed (Fig 1). The samples were enriched in Buffered *Listeria* Enrichment Broth without added antibiotics and dyes, and the enrichments subjected to Immuno Magnetic Separation (IMS) with Dynabeads anti-*Listeria* (Life Technologies, Grand Island, NY), and the beads either were directly plated onto Brilliance *Listeria* Agar or subcultured in Fraser Broth before plating onto Brilliance *Listeria* Agar. Potentially positive *L*. *monocytogenes* colonies were confirmed by PCR targeting the *hlyA* gene [26, 27, 31]. *L*. *monocytogenes* isolates were serotyped for O-Antigen by ELISA [32], and H-antigen subtyping of non-serotype 4 strains was done by PCR [33]. Lineage group identification was done by the multiplex PCR method based on the *prfA* virulence gene cluster with the primers and methods described by Ward *et al* [16]. All strains were analyzed by MLVA with 8 loci according to the method of Sperry *et al* [34, 35], as modified in our lab previously [27]. MLVA types were assigned arbitrarily based on the number of repeats of each locus, and serve as comparison purposes for these strains and those in reference [27] only. Strains of the same MLVA type have the same number of repeats at all 8 loci.

### *inlA* sequencing

The *inlA* gene from 112 *L*. *monocytogenes* isolates was amplified and sequenced. Genomic DNA was prepared using the Wizard Genomic Purification kit (Promega, Madison, WI) from cultures in Trypticase Soy Broth grown at 37°C overnight with 100 rpm shaking. Aliquots of 1.3 ml of culture were centrifuged at 13,000 rpm in a microfuge for 5 minutes, the pellets resuspended in TE (Tris-HCl, pH 8.0, 1mM EDTA) containing 10 mg/ml lysozyme, and the suspensions incubated at 37°C. Following a 30-45-minute incubation the suspensions were centrifuged at 13,000 rpm for 5 min, the supernatant removed, and the pellet lysed and treated following manufacturer’s instructions. The *inlA* gene was amplified from genomic DNA using One*Taq* Hot Start DNA polymerase (New England Biolabs, Ipswich, MA) using the protocol of Van Stelten and Nightingale [36] and the *inlA*-JK-F and R primers from Kovacevic *et al* [4]. The PCR products were purified with ExoSAP-IT and sequenced using the *inlA*-JK primer set and additional primers [36]. The additional sequencing primers were *inl*-Seq-3F: 5’- TTTCAAGTAATAAGGTGTCG-3’, *inl*-Seq-3R: 5’- AAACTAGAAACTGGGCTTAT-3’, *inl*-Seq-4F: 5’- AAGGAACGACAACATTTAGTG-3’, *inl*-Seq-4R: 5’- AAGCCTTGATAATCTACTGTT-3’, *inl*-Seq-1.5F: 5’- CAACAACTGGAGGGAACACA-3’, and *inl*-Seq-1.5R: 5’- CCTAATCTATCCGCCTGAAGC-3’. Sequencing reactions were done with Big Dye Terminator v 3.1, cleaned with the Big Dye XTerminator purification kit (Applied Biosystems), and run on an Applied Biosystems 3730 DNA Analyzer. DNA sequences were assembled and analyzed in the Lasergene 12 suite of programs (DNA Star, Madison, WI), and cluster analysis was done in BioNumerics v. 6.6 (Applied Maths, Austin, TX). *InlA* sequences were deposited into GenBank and assigned accession numbers KF728268-KF728343 and KT232270-KT232309.

## Results and Discussion

### Length of *inlA* alleles and predicted amino acid lengths

Sampling for *L*. *monocytogenes* began in January of 2012. Date and location information was recorded for each sample. At least one isolate from each positive sample was identified and saved; therefore, it is likely that some isolates are the same strain detected multiple times. For the present study, we selected an isolate from every sample positive for *L*. *monocytogenes* from the first 3 months of the survey (n = 73). Following the analysis of these 73 alleles we selected an additional 39 isolates from some of the same sampling locations as the first 73 over the next 2 years, for a total of 112 isolates and *inlA* alleles analyzed.

Of the 112 isolates 98 were Lineage I (12 of serotype 1/2b and 86 of serotype 4b), and 14 were Lineage II (all serotype 1/2a). There were 38 different MLVA types among the isolates with MLVA type 12 isolated most often. This agreed with our earlier study that showed this MLVA type was the most common in the region [27]. MLVA types clustered with Lineage with some types containing both serotypes 1/2b and 4b. Table 1 shows the summary of distribution of the predicted InlA types, S1 Table has information on each isolate.

**Table 1:** Summary of Predicted InlA lengths among 112 isolates.

Strain information				InlA type		
Serotype	Lineage	MLVA types^a^	Total isolates^b^	Intact (800 aa)	797 aa^c^	PMSC^d^ (predicted length)
1/2a	II	13	14	9	0	1 (262 aa)
1/2b	I	11	12	10	1	0
4b	I	18	86	82	7	2 (262 aa and 576 aa)

^a^ The number different MLVA types detected among the isolates of the designated serotype

^b^The total number of isolates of the designated serotype

^c^The 797 amino acid type is predicted to be functional

^d^PMSC: Premature Stop Codons

**Fig 2:**
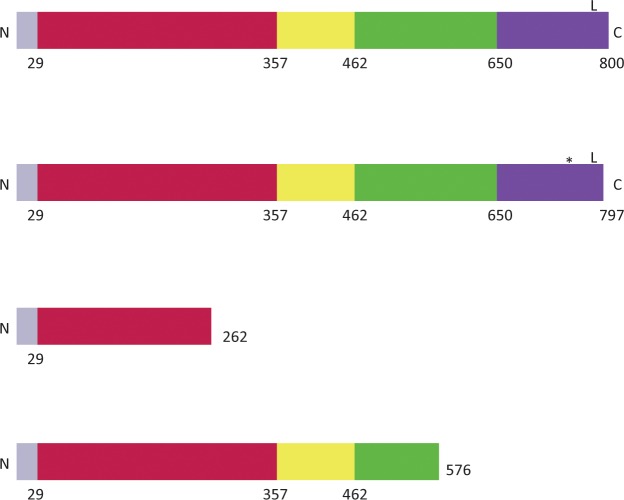
Lengths of predicted InlA proteins with functional regions labeled. The N and C termini are labeled. The final number of the right of each construct is the predicted lengths of the proteins. The InlA protein is divided into 5 regions with the first 29 amino acids (gray region) encoding a Signal Sequence. Amino acids 30–357 (red) encode a leucine rich repeat needed for attachment to the E-cadherin receptor. Amino acids 358–462 (yellow) encode an intergenic repeat. Amino acids 463–650 (green) encode the B repeats section, and amino acids 651–800 encode the Membrane Anchor region. An “L” on the Membrane Spanning region of the top two constructs indicate where the LPXTG motif is located. The region that is deleted in the 797-amino acid protein in relation to the full-length protein is indicated by an asterisk (*).

The four InlA types detected in this study are illustrated in Fig 2. Most isolates (101 or 90%) carried intact *inlA* genes predicted to encode proteins of 800 amino acids, which is divided into 5 functional domains. Only three isolates (2 of serotype 4b and 1 of serotype 1/2a) strains carried *inlA* genes with PMSCs. In one serotype 4b strain a previously described PMSC type of 576 amino acids in length (and termed in the literature “Mutation Type 12”) was found. This mutation type has been found previously in both Lineage I and II strains [5, 37]. The predicted protein would be truncated in the “B repeat” section of the protein, a region of 3 successive repeats of 70, 70, and 49 amino acids [38]. Two other strains of different lineages (a serotype 1/2a and a serotype 4b) isolated from the same watershed had a PMSC type that has not yet been described, resulting in a predicted protein of 262 amino acids that terminates within the leucine rich repeat domain that is necessary for binding to the E-cadherin receptor [13]. For these two isolates the thymidine at position 788 of the nucleotide sequence was lost, which would result in a TAA termination codon. While the two strains had *inlA* genes of the same length there were 15 SNP differences predicted to result in 6 amino acid changes between them. These SNP differences are consistent with *inlA* allelic types differing between Lineage I and Lineage II strains [12]. Orsi *et al* reported that the *inlA* genes from lineage I and II isolates are likely exposed to different evolutionary forces, and Ragon *et al*. concluded that *inlA* sequences are the result of composite evolution with the assembly of short sequences within the gene that are likely the result of multiple horizontal gene transfers [13, 39].

Another 8 isolates had a gene with a 9 nucleotide (3 amino acid) deletion in the membrane spanning region near the C terminal end of the protein (amino acids 741–743 of the intact gene). This allele type is predicted to contain a C-terminal LPXTG motif, which is the signal for the sortase proteins to anchor the protein to the peptidoglycan [40]. This allele was reported in a strain responsible for a 2002 outbreak related to deli meat, indicating the variant is still functional in virulence [41]. Kovacevic *et al*. reported on a similar 797 amino acid variant with a deletion of amino acids 738–740 in strains isolated from foods and food processing environments in British Columbia, Canada, and strains carrying this variant could invade Caco-2 cells [4]. The invasion phenotype of our strains was not tested; however, based solely on the sequence we infer that this *inlA* allele would be functional in an otherwise infection-competent strain. From an ecological standpoint, we wanted to know if this variant or any of the PMSC types persisted in this environment over time.

### Persistence of aberrant *inlA* alleles in the environment

Two PMSC types (designated alleles 3A-1, 3A-2, and 3B), and the allele with the 9-nucleotide in-frame deletion (termed allele 2) were found among the samples collected in the first 3 months, and were isolated only from the Gabilan and Carr Lake watersheds. To determine if these aberrant alleles persisted in those locations, the remaining 36 isolates in this study were from samplings of only those two watersheds from June, 2012 through May, 2014. No PMSC types were found in either watershed after that first 3-month period of sampling. Conversely, the *inlA* type predicted to encode the 797-amino acid variant was found in the same locations in March 2012, February and July 2013, and January 2014. Therefore, this allele persisted in the environment over 2 years. It was found only among Lineage I strains (7 serotype 4b isolates and 1 serotype 1/2b isolate), and these alleles were identical in nucleotide sequence. All the strains with allele 2 belonged to either MLVA type 35 or 36, which vary by 3 repeats at one MLVA locus. The collection of Allele 2 isolates represents at least three different strains, and indicate that this allele and these strains persisted in the environment over 2 years. The remaining 101 isolates (68 from samples collected January–March, 2012 and 33 from samples collected in the following 2 years) contained *inlA* alleles predicted to encode intact 800 amino acid InlA proteins. Information on all of the Allele 2 isolates is given in Table 2.

**Table 2:** Sample and isolate information for aberrant InlA types.

Sample Date	Isolate	Serotype	MLVA type	Predicted InlA Lenth (amino acids)	Watershed^a^
2012-01-11	RM15660	4b	6	262	Gabilan
2012-01-11	RM16844	1/2a	54	262	Gabilan
2012-01-11	RM15662	4b	12	576	Gabilan
2012-03-14	RM17175	4b	36	797	Tembladero
2012-03-17	RM17189	4b	36	797	Carr Lake
2012-03-17	RM17190	4b	35	797	Carr Lake
2012-03-26	RM17219	4b	36	797	Carr Lake
2012-03-26	RM17221	4b	36	797	Carr Lake
2013-02-13	RM18317	4b	36	797	Gabilan
2013-07-23	RM18326	4b	36	797	Gabilan
2014-01-28	RM18331	1/2b	36	797	Gabilan

^a^ Watersheds are shown in the map in Fig 1

### Incidence and Persistence of intact *inlA* alleles

**Fig 3:**
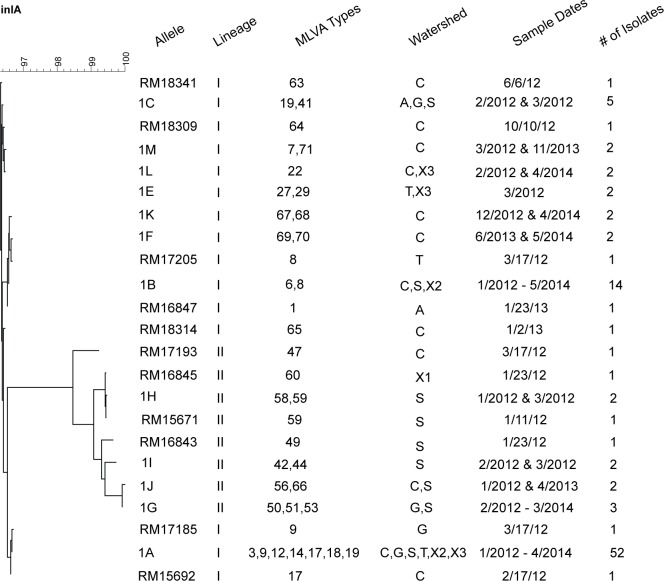
Dendrogram of intact alleles of *inlA*, and associated information. Alleles labeled with “1” and letters indicate sequences that were found in more than one isolate. Alleles labeled with “RM” and a number are unique alleles. Also listed are the Lineage information and MLVA types in which the alleles are found, watershed information, sample dates, and the number of isolates of each allele. Watershed information: A, Alisal; C, Carr Lake, G, Gabilan; S, Salinas River; T, Tembladero; X1 –X3, sites not associated with watersheds.

The complement of 101 isolates with intact *inlA* genes comprised 23 different alleles (11 of which were isolated only once), and 35 different MLVA types. Fig 3 shows a dendrogram of the alignment of the nucleic acid sequences along with the watersheds they were found, the lineages of the strains, the MLVA types, the dates, and the number of times isolated. Intact alleles isolated more than once were designated 1 + letter designation, while unique alleles are identified by RM number. The most common allele, labeled “1A,” was found in 52 isolates of serotypes 4b and 1/2b (Lineage I strains), and 7 different MLVA types, and was identical to the *inlA* sequence from strain F2365, a serotype 4b outbreak strain of *L*. *monocytogenes* responsible for a large 1985 epidemic due to contaminated cheese sourced to California. This allele was found throughout the study in 4 different watersheds, and 2 independent sites. The combination of different serotypes and MLVA types add up to at least 10 different strains carrying the 1A allele in the region with at least 7 of these strains persisting over 2 years.

**Fig 4:**
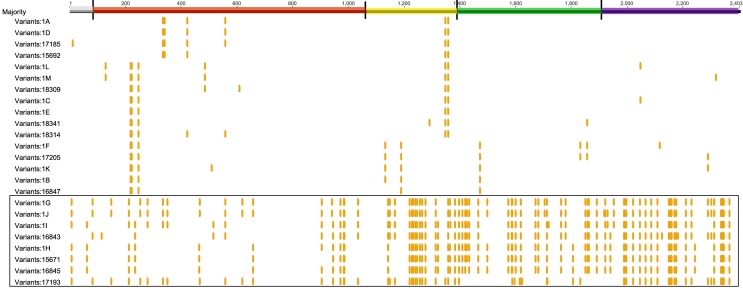
Alignment of Intact Alleles Showing SNP changes. The entire 2403 bp length of *inlA* is shown with the 5 regions as described in the caption to Fig 2. Bases 1–87 (gray) is the signal sequence, bases 88–1071 (red) is the leucine rich repeat region, bases 1072–1386 (yellow) is the intergenic region, bases 1387–1950 (green) is the B repeat region, and bases 1388–2403 (purple) is the membrane anchor region. The boxed alleles were found only in Lineage II strains, while the unboxed alleles were found only in Lineage I strains.

The second most common allele, 1B, was found in 14 isolates of Lineage I (13 serotype 4b and 1 serotype 1/2b) strains and two MLVA types. These data indicate that at least 2 strains were in the region, with at least 1 strain persisting over 2 years. The remaining alleles were found between 1–5 times, as indicated in Fig 3. The combination of different serotypes, *inlA* allele types, and MLVA types indicate that the remaining 35 isolates with intact *inlA* genes represented at least 31 different strains in the region. Many of the *inlA* alleles had matches in the GenBank database, and had been linked to human illness or were found in food samples. Alignment of the 23 intact alleles (Fig 4) indicates the SNP differences between them and a consensus sequence. The alleles break down into 2 large groupings, and these grouping were differentiated by lineage. The first grouping includes alleles 1A, 1D, 1L, 1M, 1C, 1E, 1F, 1K, and 1B along with the unique alleles in RM17185, RM15992, RM18309, RM18341, RM17206, and RM16847, which show between 5 and 7 amino acid differences from the consensus with substitutions in the receptor region, the repeat sequence3, and the membrane anchor region. Only Lineage I strains had these alleles, which were found in 98 isolates from the whole time frame of the study. The second grouping had up to 70 amino acid differences from the consensus, and includes alleles 1G, 1J, 1I, 1H, and the unique alleles in RM16843, RM15671, RM16845, and RM17193. This set of alleles was found only in Lineage II strains, and was detected in 13 strains, 12 of which were isolated in early 2012, and one was found in 2013. This clustering of *inlA* alleles based on lineage seem to agree with earlier reports, discussed above, of lineage-based differences in evolution of *L*. *monocytogenes* [13, 39]. Similar to sequences within PMSC alleles being consistent with lineage [5, 8], there is clustering among lineages with intact *inlA* alleles also. Previous work indicates that the *inlA* sequence is highly polymorphic, especially within lineage II strains [13].

There is a broad diversity of *inlA* alleles among the 3 serotypes and 2 lineages found in this region. This indicates that there are likely multiple source points for the *L*. *monocytogenes* strains found in the region with the 1A allele possibly endemic to the region. Within the number of samples in this study the indication is that *L*. *monocytogenes* encoding PMSCs were not persistent in this environment; whereas alleles encoding functional InlA do persist. Intact *inlA* alleles were found among isolates and strains from every watershed indicated in Fig 1. The three strains with PMSC’s were found in the first 3-months of the study only in the Gabilan watershed region in the mountains northeast of Salinas. The streams in this watershed can feed eventually into the Carr Lake and Tembladero watersheds, yet these truncated alleles were not found in the other regions. Our findings are different from the alleles found from foods and food processing environments in the US where 35–45% of isolates carry a truncated *inlA* gene [4]. It was reported that *L*. *monocytogenes* strains carrying intact *inlA* were able to adapt to temperature shifts from 37°C down to 4°C at a much better rate than strains carrying *inlA* genes with PMSCs [4]. Since all the isolates in the current study came from water and sediment, a persistent strain would need to withstand variations in temperature throughout the year. Furthermore, these watersheds are accessible to wildlife, and it is likely that *L*. *monocytogenes* transitions between water and wildlife in the region. A strain with a functional *inlA* would be more likely to persist among wildlife and agricultural animals as was found among ruminants [8]. The region sampled in the present study is within an agricultural region supporting many leafy green growing areas, multiple grass-fed cow-calf ranches, a beef cattle feedlot, and a dairy heifer feedlot. It is possible that these agricultural animals contribute also to the *L*. *monocytogenes* diversity found in the region. If wildlife is a primary source for the *L*. *monocytogenes* distribution in the area, then the watersheds might be more likely to carry strains with intact *inlA*. It is worth noting that due to the guidance of the Leafy Greens Marketing Agreement, leafy greens are irrigated by well water [42]. However, there is a concern that wildlife may carry pathogens from contaminated watersheds to fields, and/or that sections of agricultural fields flooded after heavy rains may be contaminated. Both scenarios illustrate potential sources of produce contamination in this leafy green growing region.

## Supporting Information

S1 Table Isolate information and inlA genotype sorted by allele type. (XLSX)
